# Active and passive soil organic carbon pools as affected by different land use types in Mizoram, Northeast India

**DOI:** 10.1371/journal.pone.0219969

**Published:** 2019-07-30

**Authors:** Uttam Kumar Sahoo, Soibam Lanabir Singh, Anudip Gogoi, Alice Kenye, Snehasudha S. Sahoo

**Affiliations:** 1 Department of Forestry, School of Earth Sciences & Natural Resource Management, Mizoram University, Aizawl, Mizoram, India; 2 Department of Forestry, Dr. Rajendra Prasad Central Agricultural University, Pusa, Samastipur, Bihar, India; 3 Indian Institute of Science Education and Research, Kolkata, Mohanpur, West Bengal, India; Sichuan Agricultural University, CHINA

## Abstract

Soil organic carbon plays an important role in the stability and fertility of soil and is influenced by different management practice. We quantified active and passive carbon pools from total soil organic carbon (TOC) in seven different land use systems of northeast India. TOC was highest (2.75%) in natural forest and lowest in grassland (1.31%) and it decreased with increasing depth in different pools of lability. Very Labile Carbon (VLC) fraction ranged from 36.11 to 42.74% of TOC across different land use system. Active carbon (AC) pool was highest in Wet Rice Cultivation (61.64%) and lowest (58.71%) in natural forest. Higher AC pools (VLC and less labile) in most land use systems barring natural forests suggest that the land use systems in the region are vulnerable to land use change and must adopt suitable management practice to harness carbon sequestration.

## Introduction

Soil organic carbon (SOC) is one of the most widely use soil quality indicator. In terrestrial ecosystems, it determines the fertility and productivity by improving the physical, chemical and biological properties, and also useful in predicting climate change and its effects [1]. Global SOC pool in the top 1 m soil is approximately 1200 to 1600 Pg (1 Pg = 10^15^ g) and 695 to 930 Pg of inorganic carbon. The amount of carbon (C) stored in soil is two times of the global biotic C pool and three times of the global atmospheric C pool [2]. The concentration of atmospheric carbon dioxide (CO_2_) may be greatly impacted with a small change in SOC pool, thus affecting the global carbon cycle [3]. It is therefore important to preserve, maintain and store SOC while addressing problems of climate change and food insecurity.

Land use/land cover change is one of the key factors which affect the soil organic carbon pool remarkably. The reason for this being the rate of input (e.g. plant litter) and rate of output (e.g. SOC mineralization) of soil organic matter (SOM) as a result of alterations in plant community and land management practice [4–5]. Tropical forests can act either as a carbon sink or source which contributes significantly in the modification of atmospheric C concentration [6]. The conversion of tropical forests into other land uses such as plantations and croplands through anthropogenic activities may act as a carbon source [7–8] leading to alterations of soil properties and processes. According to a study conducted in northern India, cultivated soils resulted in a loss of 21–36% total organic carbon as compared to uncultivated soils [9] which is a little lesser than the values (30–60%) reported in various agro-climatic regions of India [10]. When natural forests are converted to croplands, the soil structure gets disrupted enhancing the mineralization of organic matter by microbes subsequently leading to SOC loss [11]. A wide range of SOC concentration from 0.85 to 3.56% [12] with reported SOC stock of 20–40 Mg ha^-1^ in the top 0–30 cm soil depth [13] has been reported in North Eastern Himalayan Region (NEHR).

In order to understand how SOC is lost or stabilized in soil, SOC stocks in soil can be classified into different functional pools depending on their varying residence time *viz*. labile and non-labile pool. Labile pool (active pool) is the most sensitive pool available relatively in small proportion as it is easily affected by fluctuation in environmental conditions. They rapidly decompose and get oxidized easily with any changes in land use practice [14]. Another SOC pool is the non-labile pool (passive pool) which is more stable and recalcitrant fraction of SOC forming organic-mineral complexes with soil mineral and gets decomposed slowly by microbial activity [15]. Thus, the labile SOC pools serves a better indicator of soil quality to assess variations caused by land use changes [16], while the non-labile SOC pools adds to the total organic carbon stocks [17]. In an ecosystem functioning, land use change affects SOC pools to determine whether soil act as sinks or sources of C in the global C cycle [18–20].

Land use changes are one of the most important CO_2_ sources by human activities especially the conversion of natural forest to plantations and croplands in tropical and subtropics. Land use changes influence the balance between the rate of input (e.g. plant litter) and output (e.g. SOC mineralization) of soil organic matter (SOM) as a result of alterations in plant community and land management practice [4–5]. Land use changes contribute 6–39% of increase in CO_2_ emissions with profound impacts on SOC estimated at 1.5 Pg C yr^-1^ [21–22]. The decrease in soil organic matter with increase in agricultural activities has been reported in numerous studies conducted worldwide [23].

However, of late, a major land use conversion is taking place in Northeast India which is conversion of natural forest to shifting cultivation. Shifting cultivation accounts for about 85% of the total cultivated lands in Northeast India [24]. As shifting cultivation involves slashing and burning of the natural forest, it has a detrimental effect on the soil organic carbon stock and soil quality due to the huge biomass loss. Studies carried out in the region reported a significant decline in SOC stock on conversion of forests to shifting cultivation [24]. In addition, the shortened fallow cycle to meet the demands of the growing population posed a serious threat to ecology, land quality and crop production [25]. Since a vast majority of households practices this form of agriculture in Mizoram, more and more forest lands are converted to shifting cultivation leading to serious land degradation, and more so because of the steep slope condition of the state. The combination of shifting cultivation and steep slope has led to increased incidence of soil erosion making the soil highly infertile and unproductive. To address this issue, policy makers like Jhum Control Project and New Land Use Policy are trying to provide other alternatives such as agroforestry, plantation and horticulture to eradicate shifting cultivation. This effort by the policy makers have shown some progress and recently jhum lands were converted to plantation, agroforestry and other more sustainable form of land uses. Therefore, a thorough study regarding the impact of different land use type and management on the structure and content of SOM is very vital. It is evident from previous studies that change in land use system such as natural forest to agricultural land has led to a substantial decrease of both the labile and recalcitrant portion of SOM [6, 14]. This implies the possibility of loss in soil organic matter if the prevailing land use system is converted to another land use system [9].

Rate of carbon accumulation or loss in soil is greatly influenced by changes in soil and vegetation management practices [26]. SOC pool can be restored through conversion of marginal lands into restorative land uses, practice of conservation tillage with cover crops and crop residue mulch, efficient nutrient cycling with compost and manure, and other sustainable soil and water management practices [10]. In our earlier study positive rate of carbon stock change was observed when degraded lands were brought under oil palm plantation [24]. Efforts to increase SOC reservoir through carbon sequestration will nevertheless minimize global warming. Amongst different land uses types, forest plays an important role with great impact on the global biogeochemical cycles with an estimated 40% of total SOC stock stored in forest ecosystems [27]. Some studies carried out on differently aged rubber plantation in the region have shown a potential net loss of SOC stock to the tune of 67.3 Mg C ha^-1^ [28]. We hypothesized that when shifting cultivation gets converted into other form of land uses such as agroforestry and plantation, there will be an increase in SOC which in turn may have an impact on the active and passive SOC pools. However, studies and information on the distribution of SOC in different pools (active and passive) in different land use types of Mizoram, Northeast India is still limited. Therefore, the objective of this research was to quantify various SOC contents (very labile, labile, less labile and non-labile) and their relative proportions in total organic carbon (TOC) across different land use types in Mizoram, India.

## Materials and methods

### Ethics statement

The study was conducted in seven different land use systems owned by private farmers (barring natural forest). Permission was obtained from each land owner towards collection of soil samples. No rare or endangered animal was used in this experiment. Besides the study neither involved the use of wild animals nor threatened environmental systems.

### Study area

The present study was carried out in Mizoram, Northeast India. Geographically, Mizoram is located between 21°58’ N to 24°35’ N, and 91°15’ E to 93°29’ E and its total area is 21081 km^2^. The state shares southern international borders with Myanmar and Bangladesh and northern domestic borders with Manipur, Assam and Tripura. In summer, the temperature varies between 20 to 29 °C and sometimes crossing 30 °C whereas; in winter the temperature varies 7 to 22 °C. The state experiences short winter and long summer. The onset of monsoon season is from May which continues till September with an average annual rainfall of 2450 mm. The state witnessed different predominant land use/land cover types reducing forest cover of 1054 km^2^ from 2009 to 2017 where shifting cultivation (locally known as ‘Jhum’) and plantation crops (oil palm, arecanut, teak, orange, etc.) have widely replaced many of the native forests through various sponsored schemes at the cost of forest destruction and degradation To estimate the distribution of SOC stock among different land uses, we identified seven landuse types in Mizoram, *viz*. 1) Forest, 2) Agroforestry, 3) Wet Rice Cultivation, 4) Plantation, 5) Current Jhum, 6) Grassland, and 7) Jhum Fallow. A total of 38 sites were selected for the present study. Details of the site characteristics including age of the land uses, soil pH, dominant species and management practices of the land use studied were recorded with the help of the landholders and villagers and presented in Table 1.

**Table 1:** Soil characteristics and management practices of different land uses.

Land uses	Age (year)	Soil pH range	Dominant species	Management practices
Forest	41	4.47 to 4.84	*Engelhardtia spicata*, *Oryxylum indicum*, *Helicia excelsia*, *Quercus oblongata*, *Quercus floribunda*, *Rhododendron arboretum*, *Schima wallichi*	Mild anthropogenic disturbancest for occasional tree felling, frequent collection of fuelwood and other non-timber forest products
Agroforestry	10 to 17	4.59 to 4.60	*Parkia timoriana*, *Mangifera indica*, *Artocarpus heterophyllus*	Regular weeding and harvest of above ground biomass
Wet Rice Cultivation	30	5.52 to 5.56	*Oryza sativa*	Application of fertilizer.
Plantation	7 to 50	4.10 to 5.98	*Areca nut*, *Mangifera indica*, *Elaeis guineensis*, *Citrus reticulata*, *Pinus roxburghii*, *Tectona grandis*	Intercultural operations like weeding.
Current Jhum	2	4.52–4.54	*Musa accuminata*, *Carica papaya*, *Callicarpa arborea*	Annual harvest of above ground biomass, thereafter subjected to burning.
Grassland	23	4.36 to 4.92	*Eulalia trispicata*, *Imperata cylindrica*, *Cyrondon dactylon*	Subjected to annual burning.
Jhum fallow	7	4.52 to 4.56	*Musa sylvestris*	Conservation tillage and dibbling method of planting

### Soil sampling and analysis

In each land use sub type, five quadrats measuring 20 m x 20 m quadrat were randomly selected in the year 2015–2016 following ISRO-GBP/NCP-VCP protocol [29]. These quadrats were laid down in such a way that it is a representative of the land use studied. Within each quadrat, soils were collected from five points (4 in the corners and 1 in the center) at three depth classes *i*.*e*. 0–15, 15–30 and 30–45 cm respectively. The five sub-samples at each location and depth class were pooled to get one composite sample for each depth class per plot. A total of 570 samples (38 land use x 5 plots x 3 depths x 1 composite sample) were obtained for SOC analysis. The soils were mixed thoroughly and large plant debris, roots and stones were removed manually by hand. Equal number of soil samples from the same depths was collected from undisturbed plots separately for bulk density by using soil corer of known volume. In the laboratory, the soil samples were homogenized, air-dried, grounded and passed through 2 mm sieve for further analysis. For each depth, three replicates of each composite sample were analyzed.

The total carbon (TC) in the soil samples were determined by using CHNS analyzer. Inorganic soil carbon (SIC) was analyzed using dilute HCl [30]. Total organic carbon (TOC) in soil was estimated by subtracting the concentration of SIC from TC. For classifying the different pools of C according to the degrees of lability, the original Walkley and Black method is used in modified form with solutions in which the H_2_SO_4_ concentration varies at stable concentration of K_2_Cr_2_O_7_ [31]. 5, 10 and 20 ml of concentrated H_2_SO_4_ were used giving three acid-aqueous solution ratios of 0.5:1, 1:1 and 2:1 (which corresponded respectively to 12N, 18N and 24N of H_2_SO_4_). The 24N H_2_SO_4_ oxidizable C is equivalent to the standard Walkley and Black [32] method. The concentration of organic carbon (OC) determined using the three acid-aqueous solution ratios allowed separation of TOC into the following four fractions of decreasing oxidizability/lability.

1. Very labile (VLC): Organic C oxidizable under 12N H_2_SO_4_
2. Labile (LC): Difference in SOC oxidizable under 18N and that under 12N H_2_SO_4_
3. Less labile (LLC): Difference in SOC oxidizable under 24N and that under 18N H_2_SO_4_
4. Recalcitrant/ Non-labile (NLC): Residual SOC after reaction with 24N H_2_SO_4_ when compared with TOC

The very labile and labile pool may be summed up and it may be designated as active pool. Similarly, less labile and non-labile pool may be summed up and designated as passive pool. Whereas, organic carbon in rock fragments fraction is negligible; the fine soil (< 2 mm size) is of prime interest that contains the SOC. The soil samples were oven dried at 105 °C for 24 hours and passed through a 2 mm sieve to obtain the mass of fine soil. Hence, fine soil stock (FSS) in different soil depths was calculated as the product of soil layer depth with the ratio of total mass of the fine soil contained in the sample to the internal volume of the metallic core. Carbon stock in various pools (SOC stock) at different depths was computed by multiplying the respective organic carbon concentration (SOC) with measured fine soil stock (FSS) in soil samples using the formula as suggested by [33]:

$$\text{SOC}\;\text{stock}({\text{Mg}\;\text{C}\;\text{ha}}^{-1})=\text{SOC}\;(\%)\times \text{FSS}\;\left({\text{Mg}\;\text{ha}}^{-1}\right)$$

### Aboveground litter input

For aboveground litter input, five quadrats measuring 1m x 1m were laid in each land use system. Subsequently, all the litters (leaves, twigs and reproductive parts etc.) present inside the quadrat were collected and the fresh weight was taken in the field itself at monthly basis. From each plot, 100 g sample were taken to the laboratory for analysis.

### Statistical analyses

Analysis of variance (ANOVA) at 95% confidence level was analyzed taking sampling sites as replicates (random effects) and land use types as treatments (fixed effects). Tukey HSD (honestly significant difference) post hoc test was performed to indicate significant differences (p < 0.05). Figures were prepared using MS EXCEL and using SPSS for windows (IBM SPSS ver. 17.0).

## Results

### Total carbon (TC) and total organic carbon (TOC) distribution

**Fig 1:**
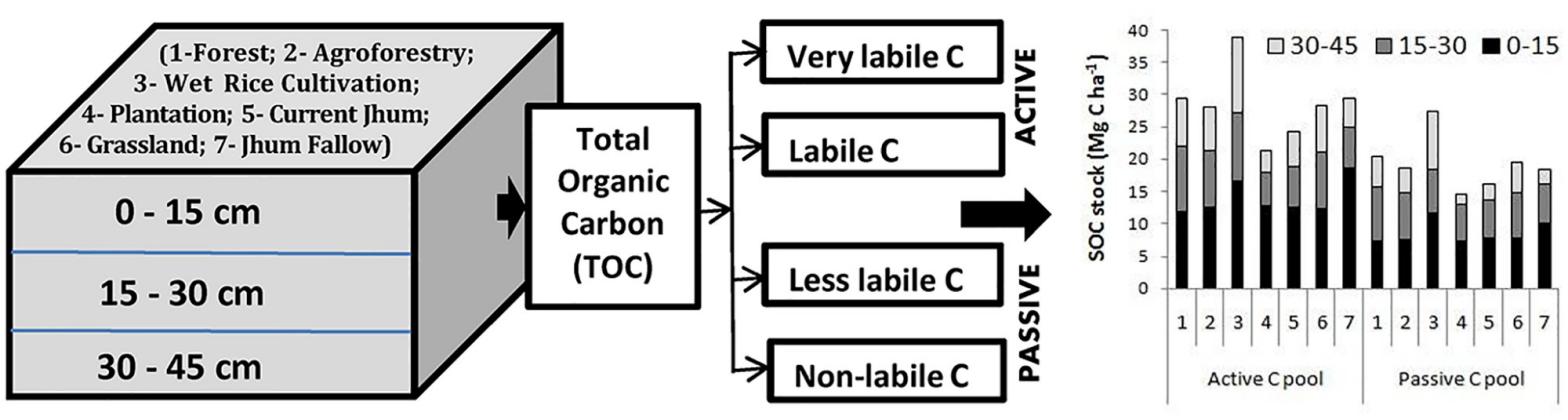
Distribution of active and passive soil carbon at three soil depths in different land use systems.

The average distribution of total carbon (TC), soil inorganic carbon (SIC) and total organic carbon (TOC) in different land use types (0–45 cm) is presented in Fig 1 and Table 2. Average fine soil stock (FSS) for 15 cm soil depth was highest in agroforestry systems (10.09 Mg ha^-1^) followed by wet rice cultivation systems (9.88 Mg ha^-1^) and the least in current jhum systems (6.08 Mg ha^-1^), however, no significant differences were observed between the studied land use systems. TC was highest in the forest (3.05%) followed by current jhum (2.19%) and the least in grassland (1.45%). SIC concentrations of these soils were small and averaged 0.14 to 0.31% under different land use types. TOC was highest in forest (2.75%)compared to other land use types, but the differences were non-significant (p<0.05), except for soil in plantation (1.38%). Average TOC content (%) in the different land use types decreased in the following order: Forest > Current Jhum > Agroforestry > Wet Rice Cultivation > Jhum Fallow > Plantation > Grassland. All the land use types under study showed higher accumulation of soil organic carbon in the top layers and decreased with increasing soil depth in different pools of lability (Table 3).

**Table 2:** Fine soil stock (FSS), total carbon (TC), soil inorganic carbon (SIC) and total organic carbon (TOC) in different land use types (0–45 cm soil depth) of Mizoram.

Land Use	FSS in 15 cm (Mg ha^-1^)	TC (%)	SIC (%)	TOC (%)
Forest	6.56±0.80^a^	3.05±0.58^a^	0.31±0.06^a^	2.75±0.52^a^
Agroforestry	10.09±0.60^a^	1.89±0.19^ab^	0.19±0.02^ab^	1.70±0.17^ab^
Wet Rice Cultivation	9.88±0.34^a^	1.78±0.06^ab^	0.16±0.02^ab^	1.62±0.07^ab^
Plantation	8.63±0.62^a^	1.53±0.13^b^	0.15±0.01^b^	1.38±0.12^b^
Current Jhum	6.08±0.98^a^	2.19±0.67^ab^	0.24±0.06^ab^	1.96±0.61^ab^
Grassland	6.67±1.56^a^	1.45±0.37^ab^	0.14±0.02^ab^	1.31±0.35^ab^
Jhum Fallow	6.37±1.69^a^	1.56±0.48^ab^	0.16±0.05^ab^	1.40±0.44^ab^

± indicates standard error of mean. Values in same column followed by different letters are significantly different (p<0.05).

**Table 3:** Soil organic carbon concentration (%) of varying lability at different soil depth classes in different land use types of Mizoram.

Land Use Types	Very Labile Carbon (VLC)			Labile Carbon (LC)		
	0–15 cm	15–30 cm	30–45 cm	0–15 cm	15–30 cm	30–45 cm
Forest	1.43±0.37 ^a^	0.89±0.11 ^a^	0.90±0.22 ^a^	0.62±0.15^a^	0.50±0.08 ^a^	0.50±0.10 ^a^
Agroforestry	0.87±0.10 ^a^	0.67±0.05^ab^	0.49±0.06 ^b^	0.39±0.05^a^	0.32±0.02^ab^	0.26±0.04 ^b^
Wet Rice Cultivation	1.29±0.03 ^a^	0.45±0.03^ab^	0.28±0.03^ab^	0.59±0.03^a^	0.22±0.02 ^b^	0.18±0.01^ab^
Plantation	0.75±0.08 ^a^	0.51±0.06 ^b^	0.40±0.33 ^b^	0.33±0.03^a^	0.25±0.03 ^b^	0.22±0.02 ^b^
Current Jhum	1.09±0.34 ^a^	0.74±0.28^ab^	0.54±0.12^ab^	0.48±0.18^a^	0.35±0.11^ab^	0.33±0.07^ab^
Grassland	0.96±0.36 ^a^	0.38±0.09 ^b^	0.22±0.02 ^b^	0.44±0.20^a^	0.19±0.03 ^b^	0.15±0.01^b^
Jhum Fallow	0.94±0.36 ^a^	0.46±0.12 ^b^	0.33±0.13^ab^	0.37±0.11^a^	0.18±0.03 ^b^	0.23±0.10^ab^
	**Less Labile Carbon (LLC)**			**Non Labile Carbon (NLC)**		
Forest	0.72±0.19^a^	0.47±0.05 ^a^	0.54±0.16 ^a^	0.71±0.14^a^	0.41±0.04 ^a^	0.54±0.24 ^a^
Agroforestry	0.37±0.04^a^	0.38±0.01^ab^	0.28±0.04^ab^	0.43±0.06^ab^	0.43±0.09 ^a^	0.20±0.03^ab^
Wet Rice Cultivation	0.53±0.01^a^	0.28±0.05^ab^	0.15±0.02^ab^	0.49±0.02^ab^	0.33±0.02 ^a^	0.09±0.02^ab^
Plantation	0.36±0.04^a^	0.31±0.03^ab^	0.24±0.02 ^b^	0.32±0.03 ^b^	0.30±0.04 ^a^	0.16±0.02 ^b^
Current Jhum	0.44±0.14^a^	0.41±0.16^ab^	0.32±0.06^ab^	0.50±0.16^ab^	0.51±0.12 ^a^	0.17±0.04^ab^
Grassland	0.39±0.20^a^	0.25±0.12 ^b^	0.14±0.01 ^b^	0.41±0.15^ab^	0.36±0.06 ^a^	0.05±0.00^ab^
Jhum Fallow	0.44±0.19^a^	0.25±0.07^ab^	0.19±0.08^ab^	0.37±0.14^ab^	0.36±0.07 ^a^	0.07±0.03^ab^

± indicates standard error of mean. Values in same column followed by different letters are significantly different (p<0.05).

### Organic carbon pools

**Fig 2:**
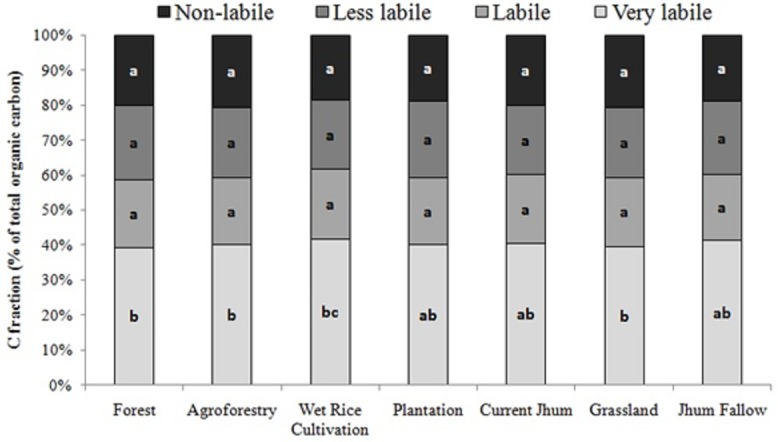
Distribution of soil organic carbon fractions of different lability (% of total organic carbon) in different land use types (0–45 cm soil depth) in Mizoram. (Letters a, b, c describe whether the proportions of different carbon fractions are significantly different or not, where proportions with same letters indicate no significance (p<0.05), within and across the land use types under study).

Soil VLC concentration in the different land uses ranged 0.22 to 1.43% along the soil profile up to 45cm depth. Soil LC and LLC contents of different land uses varied in a range of 0.15–0.62% and 0.15–0.72% respectively. Soil NLC concentration was maximum (0.71%) in 0-15cm of forest and minimum (0.05%) in 30-45cm of grassland (Table 3). The VLC fraction constituted a higher proportion of TOC with an average of 40.27% ranging from 36.11 to 42.74% in the different land use types (Table 4). The proportion of active carbon pool (VLC and LC) was higher (59.76%) than the passive carbon pool (LLC and NLC) in all the land use types (Fig 2). Active carbon pool was highest in wet rice cultivation (61.64%) and the least in forest (58.71%). All organic carbon pools were significantly (p<0.01) related to TOC (Table 5). Organic carbon fractions of different lability (VLC, LC, LLC and NLC) were also significantly correlated.

**Table 4:** Soil organic carbon concentration (%) of varying lability at in different land use types (0–45 cm soil depth) of Mizoram.

Land Use Types	Very labile	Labile	Less labile	Non-labile	Active pool	Passive pool
Forest	1.07±0.21^a^	0.54±0.09 ^a^	0.58±0.11^a^	0.58±0.11^a^	1.61±0.29 ^a^	1.13±0.22 ^a^
Agroforestry	0.68±0.07^ab^	0.33±0.13^ab^	0.34±0.02^ab^	0.34±0.02^ab^	1.07±0.25^ab^	0.70±0.07^ab^
Wet Rice Cultivation	0.67±0.03^ab^	0.33±0.02^ab^	0.32±0.02^ab^	0.32±0.02^ab^	1.00±0.08^ab^	0.62±0.01^ab^
Plantation	0.55±0.05^b^	0.26±0.02 ^b^	0.30±0.03 ^b^	0.30±0.03 ^b^	0.82±0.07 ^b^	0.56±0.05 ^b^
Current Jhum	0.79±0.25^ab^	0.39±0.13^ab^	0.39±0.12^ab^	0.39±0.12^ab^	1.18±0.38^ab^	0.78±0.23^ab^
Grassland	0.52±0.14^ab^	0.26±0.08^ab^	0.26±0.06^ab^	0.27±0.07^ab^	0.78±0.22^ab^	0.53±0.12^ab^
Jhum Fallow	0.58±0.18^ab^	0.27±0.09^ab^	0.30±0.11^ab^	0.30±0.11^ab^	0.85±0.26^ab^	0.56±0.18^ab^

± indicates standard error of mean. Values in same column followed by different letters are significantly different (p<0.05).

**Table 5:** Correlation coefficient (Pearson’s) between different organic carbon pools in soils (0–45 cm soil depth) under different land use types in Mizoram.

Variable	TOC	VLC	LC	LLC	NLC
TOC	1				
VLC	0.997**	1			
LC	0.978**	0.970**	1		
LLC	0.983**	0.980**	0.944**	1	
NLC	0.981**	0.972**	0.949**	0.948**	1

** correlarion is significant at 0.01 level (TOC- Total organic carbon; VLC- Very labile carbon; LC- Labile carbon; LLC- Less labile carbon; NLC- Non-labile carbon)

### Organic carbon stock

**Fig 3:**
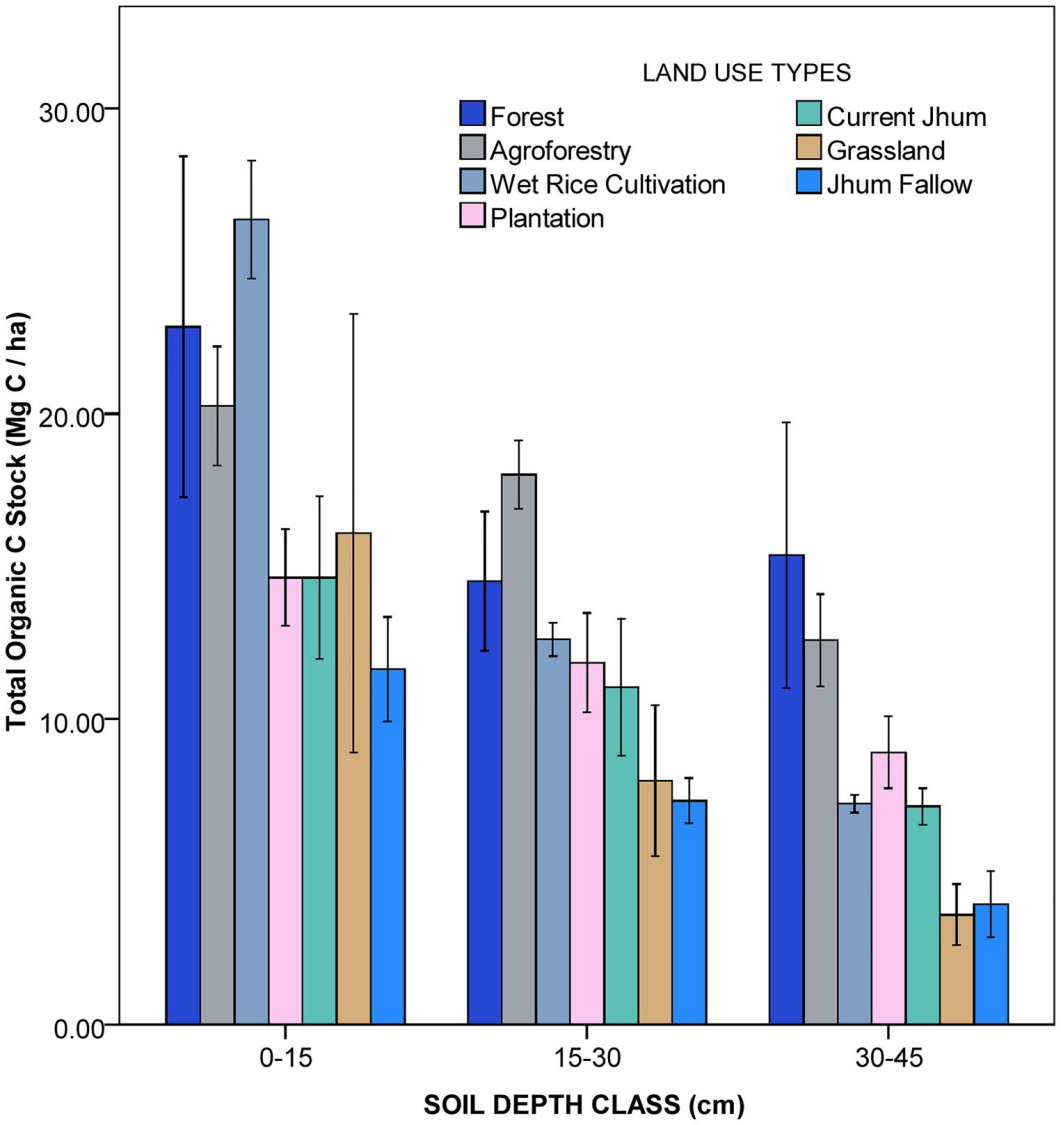
Soil organic carbon stock (Mg C ha^-1^) at different soil depth in different land use types in Mizoram.

**Fig 4:**
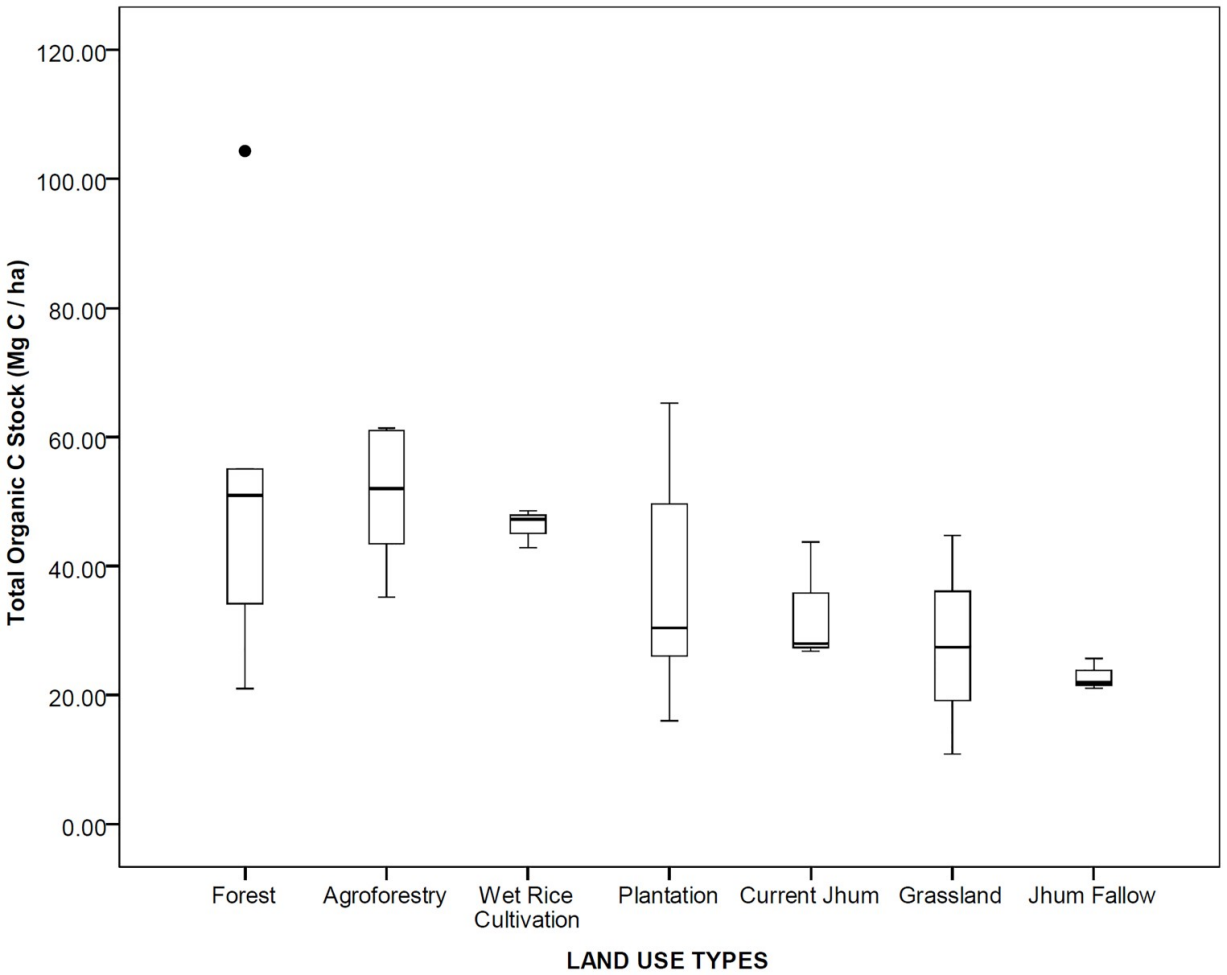
Total organic carbon stock (Mg C ha^-1^) in different land use types (0–45 cm soil depth) in Mizoram.

The soil organic carbon stock distribution at different depth classes showed a decreasing trend with increasing depth in all the land use types (Fig 3). Maximum SOC stock was stored in the wet rice cultivation (26.36 Mg C ha^-1^) at 0–15 cm soil depth. SOC storage at 15–30 and 30–45 cm soil depth classes was the maximum with 18.01 Mg C ha^-1^ and 12.59 Mg C ha^-1^ respectively in agroforestry land use type. SOC stock up to 45 cm depth soil profile was highest in forest (52.74 Mg C ha^-1^) and the least in jhum fallow (22.92 Mg C ha^-1^). SOC stock distribution in the different land use types was of the following order: Forest > Agroforestry > Wet Rice Cultivation > Plantation > Current Jhum > Grassland > Jhum Fallow (Fig 4).

### Litter input to the land use systems

**Fig 5:**
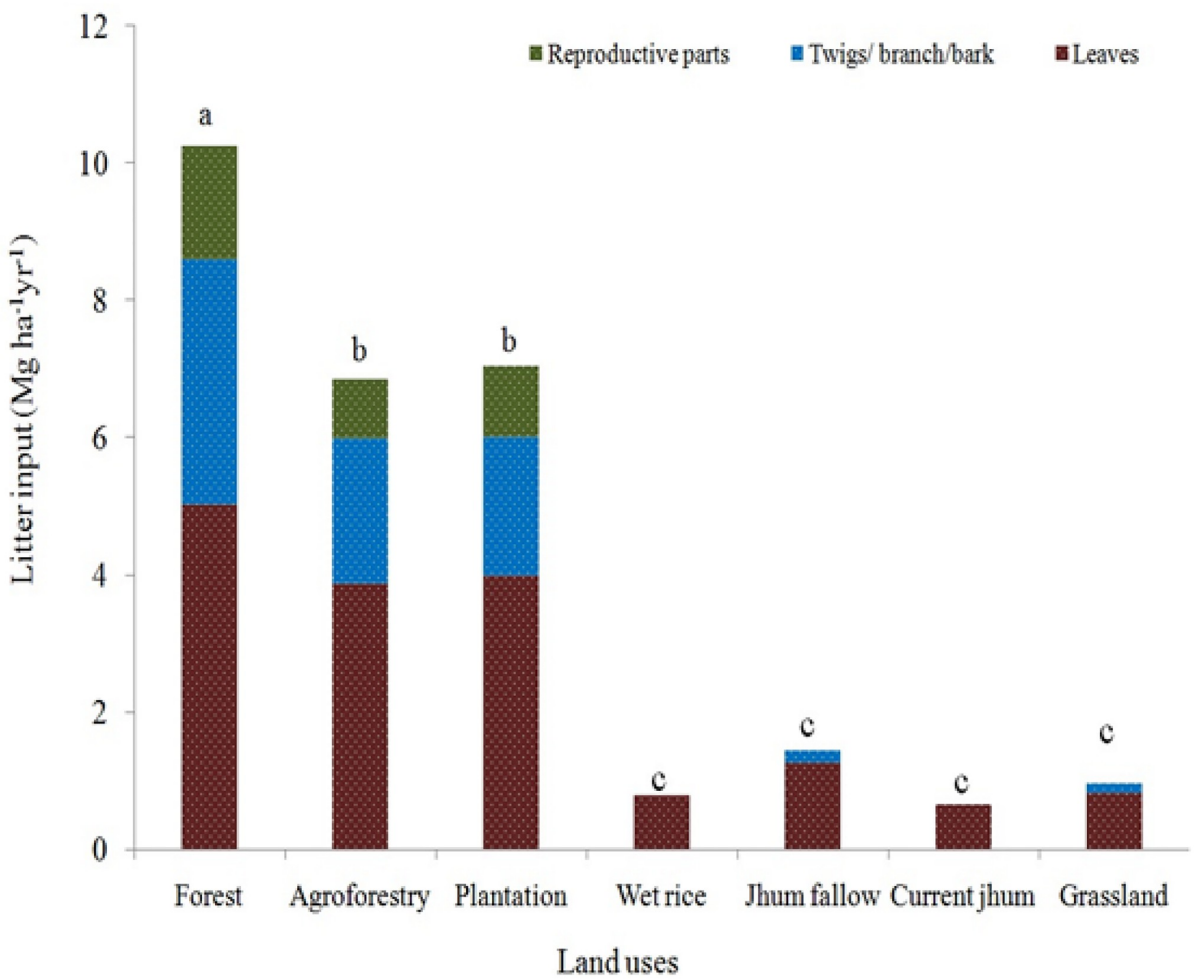
Litter inputs in different land use systems. (Different letters a, b, c indicate significant difference (p<0.05) between the different land use types under study).

Overall annual litter input was significantly higher (P<0.05) in the forest than other land use system and was in the order of forest > plantation > agroforestry> jhum fallow > grassland > wet rice cultivation and least in the current jhum (Fig 5). Among the three litter pools, leaves contributed maximally to the total annual litter inputs, and reproductive parts had least contribution to annual litter input. This was true in all land use systems. In wet rice and current jhum, leave litter pool was the only pool that contributed to the total annual litter input. Reproductive parts were absent both in jhum fallow and grassland.

## Discussion

### Total organic carbon distribution

The dynamics of carbon stock can be significantly affected by various factors such as land use history, climatic conditions, vegetation composition and management practices [34]. Analysis of 43 afforestation studies reveal that the most important factors affecting the dynamics of SOC are previous land use, climate and forest types [35]. In our present study TOC content varied across different land use types and forest has the highest TOC concentration. Soil organic carbon (SOC) content build-up under any land use type is the balance between C inputs (litter fall deposits, crop residues, root exudates, root biomass and manure) and C losses (respiration by soil organisms), determined by the residue turnover, their quality and decomposition rate [36]. A near-equilibrium between C inputs and C losses was found in undisturbed ecosystems [37]. Higher SOC content in forest land use types in our present study could be due to high level of inputs (leaf litter and root biomass) from the forest trees and its recalcitrant nature thus preventing microbial decomposition [28]. Land-use conversion can alter litter decomposition coupled with soil carbon dynamics. The increase in land-use conversion can lead to substantial gain or losses in system-level carbon [24]. Some studies have shown that the past land use can exert important control on decomposition mediated by litter quality in soil nutrient status, pH or decomposer community [38]. Therefore, litter input is a major pathway for return of dead organic matter and nutrient held in it from the aerial part of the plant community to the surface of the soil. In the present study, lesser soil TOC content in the other land use types than the forest (uncultivated) implies a considerable depletion of SOC stock by these land use types through alterations of plant species and management practices [39]. Significant differences in TOC content between forest and plantation in the present study is indicative of how SOC is influenced by the nature of crop and its management practices. These plantation land use types undergo regular intercultural operations which thereby reduces input of C to the soil. Soil tillage or other soil disturbances induced loss of C-rich macro aggregates and gain of C-depleted micro aggregates as the organic molecules are exposed mechanically [40–41]. Several studies have also shown that management practices play a vital role in determining the carbon contents in biomass and soils [42]. The SOC concentration depends on the plant productivity since management leading to input of organic matter into the soil augments the active carbon fraction [43] and continuous inputs of easily decomposable leaf litter all through the year in forests might have resulted in high value of labile C in our present study. Post burn cultivation results reduction of TOC as well as considerable variation in the proportion of different SOC pools to TOC concentration. This loss is more in the active pool (very labile and labile) than the passive pool (less labile and non-labile) of SOC [44]. In our study SOC content in current jhum was much higher than the jhum fallow and grassland. This may be attributed to the addition of burnt and partially burnt organic matter in the current jhum land during slash and burn treatment. Similar results were also reported in jhum cultivations systems of Kangchup Hills, Manipur, North-East India [45]. However, higher SOC concentration in wet rice cultivation than the jhum fallows in the present study has been ascribed to favourable water regime during the rice season. Several other studies have reported that SOC content increases with improved soil properties with the adoption of practices such as crop rotation, incorporation of plant residue and addition of composts, animal or green manure [46]. Management practices such as tillage, choice of crops and cropping systems and application of fertilizers can modify the rate of SOM decomposition by affecting the soil properties like soil moisture, soil temperature, aeration and composition [47]. These findings are in line with our results where agroforestry land use types have demonstrated high SOC concentration next to forest and current jhum land use types. SOC in the top soil (0–15 cm) in all the land use types was significantly higher than the sub-surface layers (15–30 and 30–45 cm) as also reported by several previous studies [48–49]. Loss of SOC content in the top soil as a result of land use change affects the SOC content in sub soil as the major sources of SOC (root biomass, root exudates, dissolved organic carbon) are transported from the top soil [48, 50]. Thus, SOC content of wet rice cultivation and jhum fallow in the present study exhibited low SOC content in sub soil layers as reported from studies in conventional tillage systems [51]. The decrease of SOC content with increasing soil depth is comparatively low in forest and agroforestry land use types which may be due to pronounced increase in root growth in the upper layers and absence of mechanical soil disturbance [52–53]. However, the greatly reduced SOC concentration in the deeper layers of grassland land use types may be attributed by the absence of perennial trees and a higher intensity of soil erosion [53].

### Organic carbon pools

VLC fraction of TOC was very high (average 40.27%) in all the land use types which imply a constant supply of easily decomposable litter throughout the year in the system. However, our results are in contrary with findings from other studies who reported the proportions of non-labile carbon to be more than labile carbon fractions [54]. TOC content was in a significant and positive correlation with all the labile and non-labile fractions with r values above 0.97. Results from the study are similar with studies reported [55] who recorded a high positive correlation between TOC and labile carbon (r = 0.901; p < 0.01). Active pools of TOC are more readily influenced by management practices than the passive carbon pools [55], and because of their rapid responses to environmental changes, they are identified as early indicators of soil quality [56]. Highest active carbon proportion in wet rice cultivation in the present study may be attributed by high organic matter addition and fertilizer treatments which have enhanced the root biomass yield. Root systems are reported to exudates labile carbon compounds [57]. On the contrary, forest land use types reported highest proportion of passive carbon pools in the present study. This finding may be explained by the high input of plant litter in the forest as compared to other land use types. Similar increase in passive carbon with increase in litterfall along a successional gradient has also been reported [58]. This result suggests that organic carbon in forest soils are more stable than the soils in other land use types studied if kept in continuity. This findings calls for appropriate management practices to prevent the losses of SOC through decomposition in the land use systems. Study results indicated that land use types affected both active and passive carbon pools in the different soil layers, whereby the losses in poorly managed cultivated systems may have imposed a negative impact on C sequestration [59]. Conservation tillage, incorporation of crop residues and application of manure as practiced in agroforestry land use types should be promoted for more C sequestration in agricultural soils.

### Organic carbon stock

North-East Indian landscapes are mosaic of diverse land use systems. Out of which, forest soil contributes more than 50% of the total SOC sequestration [13]. In our study, the highest SOC stock value (52.74 Mg C ha^-1^) in the forest is comparable to the previous works in the tropical evergreen forests of Mizoram [60] but it is much higher than the value of an another study on the tropical evergreen forests of Mizoram [61]. However, compared to other parts of North-East India, SOC stock value of present study is in between the range in least disturbed and mildly disturbed tropical rainforest stands reported from this region [62]. Soil SOC stock is mostly determined by physiography, altitude, bulk density, organic matter input, tree density as well as disturbance [63–65]. The higher tree density and litter inputs in the forest might have positively impacted the SOC stock in the forest. Natural forest soils are comparatively less disturbed than the soils in modified land use systems such as agroforestry, plantation and agricultural soils etc. Depth-wise SOC stock distribution showed a decreasing trend with increasing soil depth in all the land use types with an average 46.58%, 31.33% and 22.09% in 0–15, 15–30 and 30–45 cm soil depth respectively within the 0–45 cm soil profile. The decreasing trend of SOC stock along soil depth has been observed in many studies [64]. According to Rumpel and Kogel-Knabner, radio carbon age of SOC increases with depth, while SOC and C/N ratios decrease with increasing depth [66]. Highest proportion of SOC in the top soil (0–15 cm) was reported in wet rice cultivation (56.91%) followed by grassland (55.04%) in accordance to the SOC vertical distribution pattern reported by other studies across the world [67–68] implying the vulnerability of these land use types to SOC stock losses under soil and water erosion. Study results indicated that agroforestry land use types stored highest SOC stock next to forest which was similarly reported by other studies [69–70] as result of greater species diversity, composition and low intensity of soil disturbance compared to agricultural systems [71]. Evaluation of SOC storage potential and improving the biological cycle of ecosystems to maintain equilibrium in fixation and storage of carbon is thus important [72].

## Conclusions

Within the 0–45 cm soil profile, TOC content and SOC stocks were all higher in forest than the other land use types (agroforestry, wet rice cultivation, plantation, current jhum, grassland, jhum fallow) in the present study, indicating the depletion of TOC with land use changes. A positive correlation was observed between the various labile carbon fractions and TOC in the soil. The proportion of active carbon pool (VLC and LC) was higher (59.76%) than the passive carbon pool (LLC and NLC) in all the land use types, suggesting that the accumulated carbon could be easily lost following land use changes. However, forest land use types reported highest proportion of passive carbon pools indicating more stable nature of the accumulated soil organic carbon. Results suggest that both the active and passive C pools are affected by land use types in different soil layers, and the dynamics of SOC turnover in the soil profile with land use management practices should be paid more attention to harness the C sequestration potential of soil efficiently. Increased addition of plant residues coupled with less disturbance in forest compared to other land uses led to higher SOC stock in the former indicating its role in maintaining and sustaining soil health. The present research work gave an insight how soil carbon build-up could be improved if provided with proper management practices where policy decisions should embrace multidisciplinary and multiregional approach.

## Supporting information

S1 File SOC and litter data of different land use types in Mizoram. (XLSX)
